# Retrieval-Extinction and Relapse Prevention: Rewriting Maladaptive Drug Memories?

**DOI:** 10.3389/fnbeh.2020.00023

**Published:** 2020-02-20

**Authors:** Eloise J. Kuijer, Antonio Ferragud, Amy L. Milton

**Affiliations:** ^1^Department of Psychology, University of Cambridge, Cambridge, United Kingdom; ^2^Leiden University Medical Centre, Leiden University, Leiden, Netherlands

**Keywords:** memory reconsolidation, extinction, retrieval-extinction, addiction, rat

## Abstract

Addicted individuals are highly susceptible to relapse when exposed to drug-associated conditioned stimuli (CSs; “drug cues”) even after extensive periods of abstinence. Until recently, these maladaptive emotional drug memories were believed to be permanent and resistant to change. The rediscovery of the phenomenon of memory reconsolidation—by which retrieval of the memory can, under certain conditions, destabilize the previously stable memory before it restabilizes in its new, updated form—has led to the hypothesis that it may be possible to disrupt the strong maladaptive drug-memories that trigger a relapse. Furthermore, recent work has suggested that extinction training “within the reconsolidation window” may lead to a long-term reduction in relapse without the requirement for pharmacological amnestic agents. However, this so-called “retrieval-extinction” effect has been inconsistently observed in the literature, leading some to speculate that rather than reflecting memory updating, it may be the product of facilitation of extinction. In this mini review article, we will focus on factors that might be responsible for the retrieval-extinction effects on preventing drug-seeking relapse and how inter-individual differences may influence this therapeutically promising effect. A better understanding of the psychological and neurobiological mechanisms underpinning the “retrieval-extinction” paradigm, and individual differences in boundary conditions, should provide insights with the potential to optimize the translation of “retrieval-extinction” to clinical populations.

## Introduction

Addiction is a chronic, relapsing disorder characterized by loss of control over drug use, high motivation for drug, and persistence in drug use despite adverse consequences (American Psychiatric Association, 2013). Those who become addicted show a high propensity to relapse following periods of abstinence. Re-exposure to previously drug-associated cues is one major precipitant of relapse: people, places, and paraphernalia repeatedly paired with drugs become conditioned to the drug high in a pavlovian manner, and these pavlovian conditioned stimuli (CSs) subsequently induce relapse (de Wit and Stewart, 1981).

Drug-associated CSs influence relapse through at least three psychologically and neurobiologically dissociable processes (Milton and Everitt, 2010). Until recently, these maladaptive CS-drug memories were believed to be permanent and resistant to change. However, following the rediscovery of memory reconsolidation (Nader et al., 2000) interest grew in exploiting this process to develop new forms of treatment for mental health disorders including addiction. One such strategy would be pharmacological disruption of drug memory reconsolidation with the administration of amnestic agents (for review, see Milton and Everitt, 2010). Here, we focus on an alternative strategy aiming to capitalize on the hypothesized updating function of reconsolidation; reactivating a memory and introducing “CS-no US” information through the procedure known as “extinction within the reconsolidation window” or “retrieval-extinction.” Due to the relative paucity of drug memory retrieval-extinction studies in the literature, we will extrapolate general principles from retrieval-extinction studies of both fear and drug memories, focusing on the influence of individual differences.

## Retrieval-Extinction as a Non-pharmacological Memory Interference Method

A potential limitation of pharmacological approaches to target memory reconsolidation is the requirement for amnestic agents. Although drugs such as propranolol, the β-adrenergic receptor antagonist used in many reconsolidation studies, are safe to use in humans, many amnestic agents (e.g., protein synthesis inhibitors) are less well-tolerated. Consequently, there has been great interest in capitalizing on the hypothesized role of reconsolidation in memory updating (Lee, 2009) with the use of “retrieval-extinction” procedures.

“Retrieval-extinction” was first described for pavlovian fear memories, and involves reactivating the memory in a brief re-exposure session, followed by a separate prolonged re-exposure/extinction session after a short delay (typically 10–60 min, but theoretically within 3–4 h of the opening of the “reconsolidation window”). The retrieval-extinction procedure persistently attenuates recovery of fear memories in both rats (Monfils et al., 2009) and humans (Schiller et al., 2010), although this has not been universally replicated (e.g., see Luyten and Beckers, 2017).

Shortly after the discovery of retrieval-extinction, a seminal article (Xue et al., 2012) showed that retrieval-extinction could reduce drug-seeking in rodents trained on cocaine- or opiate-conditioned place preference (CPP) or intravenous cocaine self-administration. Furthermore, retrieval-extinction was shown in the same study to reduce craving elicited by heroin CSs in human outpatient heroin abusers. This has a potentially profound impact on addiction treatment, as a relatively minor adjustment to prolonged exposure therapy greatly improved treatment outcomes. Consequently, there has been intense research interest in retrieval-extinction from both preclinical and clinical addiction researchers.

Reductions in CPP following the retrieval-extinction procedure have been replicated with cocaine (Sartor and Aston-Jones, 2014) and morphine (Ma et al., 2012). Retrieval-extinction also reduces alcohol-seeking in rats (Millan et al., 2013; Willcocks and McNally, 2014; Cofresí et al., 2017) and nicotine-seeking in human smokers (Germeroth et al., 2017). However, despite its efficacy in reducing drug-seeking, there remains a lack of definitive evidence that retrieval-extinction for drug memories depends critically upon memory-updating and reconsolidation mechanisms, and not the facilitation of extinction. In several studies where retrieval-extinction effectively reduced one measure of drug-seeking, it was ineffective at reducing other measures: it did not prevent spontaneous recovery of morphine CPP 4 weeks post-intervention (Ma et al., 2012) and it did not retard the reacquisition of alcohol-seeking, as would be expected if the original cue-alcohol memory had been erased (Willcocks and McNally, 2014). Furthermore, the finding that extinction training *prior* to memory reactivation reduces subsequent alcohol-seeking contradicts the hypothesis that memory destabilization is critical for the retrieval-extinction effect (Millan et al., 2013). This is consistent with our previous report that drugs that block fear memory destabilization do not prevent the reduction in fear produced by the retrieval-extinction procedure (Cahill et al., 2019).

However, it may be premature to conclude that retrieval-extinction simply represents the facilitation of extinction that does not engage in memory reconsolidation mechanisms. Some molecular evidence suggests that retrieval-extinction recruits immediate early genes associated with memory reconsolidation, at least for fear memories (Tedesco et al., 2014) and that antagonism of L-type voltage-gated calcium channels, which are necessary for memory destabilization (Suzuki et al., 2008) prevents the reduction in subsequent responding normally observed following retrieval-extinction for a food-associated CS (Flavell et al., 2011). These apparently conflicting findings are difficult to reconcile, but we propose that individual differences may determine whether reconsolidation or extinction mechanisms are engaged under a given set of experimental conditions. In turn, this may account for the inconsistent reports of retrieval-extinction in the literature.

## The Influence of Individual Differences on the Efficacy of Retrieval-Extinction

Individual differences pose a potential challenge to the translation of retrieval-extinction to the clinical situation. A relatively understudied phenomenon in retrieval-extinction, individual differences in the acquisition of extinction influence the efficacy of retrieval-extinction for preventing the recovery of fear memories (Shumake et al., 2018) and in turn, the capacity for fear extinction learning correlates with CO_2_ reactivity and orexin expression in the lateral hypothalamus (Monfils et al., 2019). To date, there have been no studies examining the impact of these mechanisms on the retrieval-extinction of appetitive memories, but drawing on findings from the fear literature, we consider three factors that are likely to influence retrieval-extinction for drug memories: individual differences in reconsolidation boundary conditions, the attribution of incentive value to appetitive cues, and the influence of stress on mnemonic processes.

### Individual Differences in Boundary Conditions

Not all instances of memory retrieval lead to memory reconsolidation; instead, there are hypothesized “boundary conditions” that determine whether a retrieved memory destabilizes and reconsolidates. There is extensive evidence that memory destabilization depends upon a “mismatch” between what is expected and what actually occurs, formalized as “prediction error” (Pedreira and Maldonado, 2003; Pedreira et al., 2004; Sevenster et al., 2012, 2013, 2014; though see Yang et al., 2019, for a discussion of whether uncertainty may also induce memory destabilization). The relationship between prediction error and memory lability is not monotonic, however, as extensive prediction error—for example, during extended periods of reinforcer omission—leads not to reconsolidation of the original memory, but rather the consolidation of new extinction memory, and thus extinction learning. The relationship between reconsolidation and extinction has been extensively investigated for fear memories, with converging evidence showing that the two mnemonic processes are separated by a “limbo” period in which the original memory becomes again insensitive to disruption (Flavell and Lee, 2013; Merlo et al., 2014, 2018; Sevenster et al., 2014; Cassini et al., 2017). To date, this has been studied at the population level with strong conditioning parameters, which may mask individual variability. For drug memories, where individual drug use histories show greater variability, it may be hypothesized that the extent of prediction error required to engage reconsolidation, limbo and extinction mechanisms may differ between individuals. Thus, considering the widely accepted boundary conditions of memory strength and age (Suzuki et al., 2004; Kwak et al., 2012), the extent of re-exposure required for reactivating a cue-drug memory may individually vary.

### Individual Differences in Attribution of Incentive Value to Cues

An increasingly large body of research has characterized how individual differences in the attribution of incentive value to drug-associated CSs influence subsequent drug self-administration and relapse (see Robinson et al., 2018, for review). There is variation in the degree to which individuals are attracted to discrete CSs associated with reward (“sign-tracking”) as compared to the location of the reward itself (“goal-tracking”), usually measured by a pavlovian conditioned approach using an autoshaping procedure (Meyer et al., 2012). These behaviors are hypothesized to reflect endophenotypes correlated with differences in dopaminergic signaling within the motivational circuitry (Flagel et al., 2011) and differential reliance on model-based (goal-directed) and model-free (habitual) motivational systems (Lesaint et al., 2015). There is also evidence that goal-trackers condition more readily than sign-trackers to contextual cues predictive of reinforcement (Morrow et al., 2011; Saunders et al., 2014), although this has not been universally replicated (Vousden et al., in press).

Sign-trackers and goal-trackers appear to learn differentially about discrete and contextual cues. This may influence whether they perceive the retrieval-extinction procedure to be the same as the previous learning experience (favoring reconsolidation updating) or as a different learning experience (favoring the formation of a new extinction memory). We speculate that sign-trackers and goal-trackers may attribute the retrieval-extinction experience to different “latent causes” (Dunsmoor et al., 2015). Considering that sign-trackers also appear to be resistant to pavlovian extinction (Ahrens et al., 2016), the relative paucity of studies of the influence of these endophenotypes on retrieval-extinction is surprising. Those that have been conducted used a slightly different procedure, classifying rats as “orienters” and “non-orienters” to pavlovian CSs, which are broadly similar to sign-tracking and goal-tracking. Both groups showed reduced spontaneous recovery of fear memory (Olshavsky et al., 2013), but when the appetitive CS-reward memory was targeted for retrieval-extinction, only the orienters/sign-trackers showed reduced appetitive responses (Olshavsky et al., 2014). This may suggest a shift in the boundaries between reconsolidation, limbo and extinction, such that the same re-exposure session may have induced reconsolidation-based updating in the sign-trackers, but limbo or extinction in the goal-trackers, reflecting the increased sensitivity of goal-trackers to contextual cues (including interoceptive, temporal cues) that distinguish the retrieval session from previous learning.

### Individual Differences in the Effects of Stress on Extinction

The discrepancies within and between studies of “retrieval-extinction” could potentially be explained by different individual stress levels during either the reconsolidation or the extinction session(s), whether stress is induced through re-exposure to an aversive CS or by frustration by the omission of an appetitive drug reward (e.g., Ginsburg and Lamb, 2018). The effect of stress is usually to impair reconsolidation, as has been reviewed previously (Akirav and Maroun, 2013), so here we focus on the effects of stress on extinction.

The relationship between stress and extinction is complicated, depending critically upon the degree and timing of stress relative to extinction learning and retrieval. Mimicking stress through the administration of low doses of exogenous glucocorticoids enhances, whilst high doses impair, consolidation (Roozendaal, 2003). This depends upon the activation of glucocorticoid receptors in the amygdala, which modulates both the acquisition and consolidation of fear extinction (Yang et al., 2006) in an NMDA receptor-dependent manner (Yang et al., 2007). These dose effects of glucocorticoids depend critically on the receptors activated, with glucocorticoid receptors and mineralocorticoid receptors having differential roles in contextual fear extinction (Ninomiya et al., 2010; Blundell et al., 2011).

Timing of stress relative to extinction learning or retrieval determines whether stress enhances or impairs the behavioral expression of the extinction memory, as articulated in the Stress Timing affects Relapse (STaR) model (Meir Drexler et al., 2019). This model proposes that stress or glucocorticoid administration prior to extinction learning increases consolidation of the extinction memory such that it is less context-specific (de Quervain et al., 2011), and that post-extinction stress or glucocorticoid administration also enhances its consolidation, but in a context-dependent manner. By contrast, stress or glucocorticoid administration immediately before an extinction retrieval test impairs extinction retrieval, leading to increased fear. However, though the STaR model (Meir Drexler et al., 2019) is well supported by evidence from human studies of contextual fear, the evidence from discrete fear learning (summarized in Table 1) is not always consistent with stress enhancing extinction consolidation. The studies presented here show generally that stress, either behaviorally induced or by corticosterone administration, has a neutral or even detrimental effect on the distinct phases of extinction and retrieval. However, in the acquisition or consolidation of extinction in contextual fear, a few studies show enhancing potential. Importantly, for the extinction of maladaptive appetitive drug associations, no studies indicate enhancing the therapeutic potential of stress. The contrast of stress effects between different types of memory likely reflect the different effects of stress hormones in the hippocampus, which is required for contextual fear learning, and the amygdala, required for both contextual and discrete fear learning (McEwen et al., 2016).

**Table 1:** Modulation of different phases of extinction by behavioral stress, glucocorticoid administration, and secondary interventions.

Type of memory	Mnemonic phase	Effect	Method of stress induction	References	Secondary intervention	Total effect	References
CS-US fear conditioning and extinction	Acquisition or consolidation of extinction	Impaired	Behavioral	Izquierdo et al. (2006), Akirav and Maroun (2007), Yamamoto et al. (2008)^2^, Akirav et al. (2009)^1^, Farrell et al. (2010), Knox et al. (2012b), Maroun et al. (2013), Keller et al. (2015) and Sawamura et al. (2016)	Dexamethasone Metyrapone Infralimbic lesion Diazepam D-cycloserine D-cycloserine	Rescued Exacerbated Impaired Rescued No effect Rescued	Sawamura et al. (2016) Keller et al. (2015) Farrell et al. (2010) Akirav and Maroun (2007) Akirav et al. (2009)^1^ Yamamoto et al. (2008)^2^
		No effect	Behavioral	Miracle et al. (2006); Garcia et al. (2008); Wilber et al. (2011) and Knox et al. (2012a)			
			CORT	Wang et al. (2014)			
	Retrieval of extinction	Impaired	Behavioral	Miracle et al. (2006), Garcia et al. (2008), Farrell et al. (2010), Wilber et al. (2011), Knox et al. (2012a), Deschaux et al. (2013), Maroun et al. (2013) and Xing et al. (2014)	Fluoxetine Infralimbic lesion	Rescued Rescued	Deschaux et al. (2013)^3^ Farrell et al. (2010)
		No effect	CORT	Wang et al. (2014)			
Context-fear conditioning and extinction	Acquisition or consolidation of extinction	Impaired	Behavioral	Akirav and Maroun (2007), Yamamoto et al. (2008)^2^ and Akirav et al. (2009)	D-cycloserine Diazepam	Rescued Rescued	Yamamoto et al. (2008)^2^ and Akirav et al. (2009) Akirav and Maroun (2007)
			CORT	Gourley et al. (2009)	Mifepristone	Mimicked	Gourley et al. (2009)
		No effect	Behavioral	Knox et al. (2012a)			
		Enhanced	Behavioral	Kirby et al. (2013)			
			CORT	Cai et al. (2006), Abrari et al. (2008) and Blundell et al. (2011)			
Instrumental conditioning for drug reward and cued extinction	Acquisition or consolidation of extinction	No effect	Behavioral	Eagle et al. (2015) and Manvich et al. (2016)			
	Retrieval of extinction	No effect	Behavioral	Eagle et al. (2015)^4^			
		Enhanced reinstatement	Behavioral	Erb et al. (1998)^5^, Graf et al. (2013)^6^ and Manvich et al. (2016)^4^	ADX ADX + CORT	Rescued Reinstated	Erb et al. (1998)^5^ and Graf et al. (2013)^6^ Erb et al. (1998)^5^
			CORT	Graf et al. (2013)^6^	Mifepristone	No effect	Graf et al. (2013)

*Abbreviations: ADX, adrenalectomized; CORT, corticosterone. Note that this table includes only rodent studies. Other exclusions consist of: studies that applied the stress before conditioning when this had a significant effect on conditioning, e.g., studies using early life stress, or when they did not provide any conditioning data as this renders it impossible to conclude on the effects on extinction alone. For the effects on retrieval of extinction as determined by performance during reinstatement, only studies were included which targeted the stress specifically to the extinction session, and not to the reinstatement session. Also, articles that did not provide controls for stress/CORT induction were excluded. No articles on retrieval of extinction within contextual fear, nor articles which used CORT to induce stress in instrumental conditioning were found after these exclusions. Specific excluded articles, as it is beyond the scope of this table: effect of strain in mice (Brinks et al., 2009); diurnal changes in corticosterone (Woodruff et al., 2015); gender (Baran et al., 2009); exposure to novel context (Liu et al., 2015); conditioning using conditioned place preference (Leão et al., 2009; Taubenfeld et al., 2009; Karimi et al., 2014; Meng et al., 2014; Ebrahimian et al., 2016; Taslimi et al., 2018). The severity of behavioral stress induction, nor CORT dose showed no clear effect and is thus for clarity not included*.

*^1^This study was the only one in the CS-US category where conditioned taste aversion was used to establish the CS-US association. All others used classical cue-fear conditioning, where a fear-related US, typically an electrical shock, is paired to a CS, typically a tone*.

*^2^This study used classical cue-fear conditioning but used only the context for extinction*.

*^3^The fluoxetine was given for 21 days after extinction. The behavioral stress consisted of elevated platform prior to retrieval. The decrease in freezing could also be interpreted as an enhancing effect of fluoxetine on the consolidation of extinction, rather than retrieval, or could be ascribed to the general anxiolytic effects of fluoxetine*.

*^4^Reinstatement was not cocaine-primed*.

*^5^Reinstatement was cocaine-primed*.

*^6^This effect was only observed when animals received a priming dose of cocaine vs. saline prior to the reinstatement test*.

To our best knowledge, the effects of stress have not been systemically investigated in the context of retrieval-extinction. Based on the STaR model (Meir Drexler et al., 2019) it may be possible to optimize retrieval-extinction using well-timed glucocorticoid administration. However, based on Table 1, we would only expect this to work for contextual fear extinction, and to have a limited or even detrimental effect for appetitive memories, regardless of whether retrieval-extinction is mediated by an extinction or reconsolidation mechanism. Importantly, differences in stress state would be predicted to affect the acquisition, consolidation, and retrieval of extinction, thus potentially explaining the large variation between retrieval-extinction studies.

## Optimizing Retrieval-Extinction for the Disruption of Drug Memories

Considering the influence of these individual differences on retrieval-extinction, how might the procedure be individually optimized?

### Optimizing Memory Reactivation

Reconsolidation deficits are highly selective to the reactivated memory (Dębiec et al., 2006; Doyère et al., 2007), which could limit the efficacy of reactivation based on the presentation of CSs. Furthermore, individual differences exist in attention and engagement with CSs (Meyer et al., 2012), which could account for differences in the efficacy of retrieval-extinction, such as those seen with appetitive memories (Olshavsky et al., 2014).

US presentation can also be used to reactivate memories. It was first shown in studies of fear memory that unsignalled re-exposure to footshock could destabilize the fear memory and make it susceptible to disruption with protein synthesis inhibition (Dębiec et al., 2010). Similarly, re-exposure to the US induced susceptibility to retrieval-extinction, and led to reductions in fear to all CSs associated with the US, rather than individual CS-US associations (Liu et al., 2014). US-based reactivation has also been shown to extensively reduce reactivation-induced CREB expression, compared to CS-based reactivation (Huang et al., 2017).

A similar US-based reactivation approach has been used in studies of drug memory reconsolidation. In rats extensively trained to self-administer cocaine, reactivation of the drug memory through experimenter-administered injections of cocaine, followed by drug memory extinction, reduced reinstatement, spontaneous recovery and renewal (Luo et al., 2015). Importantly, the retrieval-extinction effect was also observed when instead of cocaine, the stimulant methylphenidate was administered. As noted by the authors (Luo et al., 2015), this overcomes the difficult ethical issue of administering an illegal drug to a patient who is trying to maintain abstinence. However, these findings do raise questions regarding the mechanism by which US-based reactivation occurs. It may reactivate a “US engram” in the brain, propagating destabilization along the network of associated CSs. Alternatively, US exposure could lead to experiencing interoceptive cues that reactivate the drug memory which may account for the increased efficacy of US-based reactivation procedures. A specific test of the latter hypothesis would be to determine whether drug isoforms that do not cross the blood-brain-barrier—and so could only produce central effects through the detection of peripheral interoceptive cues—would be as effective in reactivating the memory as drugs that do cross the blood-brain-barrier. To our knowledge, this remains to be investigated.

### Optimizing Extinction

The fact that there are no standardized procedures to destabilize memory makes the interpretation of studies failing to replicate retrieval-extinction difficult. Although memory destabilization—at least for pavlovian memories—is thought to depend on inducing a “violation of expectations” or “prediction error” (Pedreira et al., 2004; Sevenster et al., 2013, 2014), it is widely accepted that the relationship between prediction error and memory destabilization is complex. As noted above, re-exposure to a single previously fear-associated CS will induce memory reconsolidation, but greater re-exposure (with more prediction error) leaves the original memory intact and instead promotes the consolidation of an extinction memory after a “limbo” period (Lee et al., 2006; Merlo et al., 2014, 2018). Therefore, the relationship between prediction error and memory destabilization is not linear, leading some to hypothesize that destabilization may instead be driven by the attribution of an unexpected experience to the same underlying “latent cause” as has been experienced in the original consolidation of the memory (Dunsmoor et al., 2015; Gershman et al., 2017). The difficulty in empirically determining whether an experience is attributed to the same or different latent cause—which could also differ between individuals—leads us to hypothesize that the failures to replicate the retrieval-extinction effect may be due to engaging the facilitation of extinction, rather than destabilization of the original memory. One major challenge in distinguishing between these two accounts of retrieval-extinction is the reliance on a single behavioral readout. We have previously argued (Cahill and Milton, 2019) that corroborating molecular evidence would be useful in this respect.

Certainly, our own data are more consistent with a “facilitation of extinction” account of retrieval-extinction. We observed (Cahill et al., 2019) the retrieval-extinction effect for fear memories despite behavioral manipulations of prediction error and selective pharmacological blockade of the D_1_-subtype of dopamine receptor, which is required for memory destabilization (Merlo et al., 2015). Furthermore, considering studies showing facilitation of extinction following exposure to a novel environment (de Carvalho Myskiw et al., 2013; Liu et al., 2015), at least some of the published putative retrieval-extinction effects could be due to the facilitation of learning by a proximal behavioral experience. This phenomenon, in which novelty exposure facilitates subsequent learning, is known as “behavioral tagging” (Moncada and Viola, 2007; Moncada et al., 2011). One test of the “facilitation of extinction” account of retrieval-extinction would be to expose animals to a novel context prior to extinction training, rather than a memory reactivation session; if the “retrieval-extinction” effect persists despite a lack of memory reactivation, this would cast doubt on the reconsolidation-based account of the phenomenon.

Determining whether retrieval-extinction depends upon reconsolidation or extinction mechanisms is of great potential importance in optimizing this therapeutic strategy. For example, if dependent primarily on extinction mechanisms, then it may be possible to facilitate retrieval-extinction further with the administration of drugs such as the glutamate receptor partial agonist D-cycloserine (Das and Kamboj, 2012). However, the use of drugs to enhance retrieval–extinction may reduce the non-pharmacological appeal of the intervention. Alternatively, if individual differences determine whether reconsolidation-update or extinction mechanisms are engaged by the retrieval-extinction procedure, then identification of these differences—for example, by classifying individuals as sign-trackers or goal-trackers, or determining stress reactivity—could be used to optimize the retrieval-extinction procedure by targeting the dominant mnemonic process in each individual.

## Conclusions

Although the mechanisms underlying retrieval-extinction remain unclear, and retrieval-extinction has not been universally replicated, this process has great potential for the treatment of drug addiction. Understanding the contribution of individual differences to the boundary conditions underlying reconsolidation, limbo, and extinction, and how these interact with factors such as the attribution of incentive value to appetitive stimuli and stress, may provide insight into the apparent inconsistencies in the literature, and guide future optimization of retrieval-extinction for clinical use.

## Author Contributions

EK, AF, and AM wrote and critically edited the manuscript.

## Conflict of Interest

The authors declare that the research was conducted in the absence of any commercial or financial relationships that could be construed as a potential conflict of interest.
